# Clinical analysis of patients with systemic lupus erythematosus complicated with liver failure

**DOI:** 10.1007/s10067-023-06524-9

**Published:** 2023-02-16

**Authors:** Lili Zhang, Ling Yin, Wenliang Lv, Yitong Wang, Yang Liu, Chunyan Gou, Jianhua Hu, Xiaojun Wang

**Affiliations:** 1 Integrated Traditional Chinese and Western Medicine Center, Beijing Youan Hospital, Capital Medical University, Beijing, 100069 China; 2 Department of Infectious Diseases, Guang’anmen Hospital, China Academy of Chinese Medical Sciences, Beijing, 100053 China

**Keywords:** Autoimmune hepatitis, Hormone therapy, Liver failure, Systemic lupus erythematosus

## Abstract

The objective of this study is to analyze and summarize the characteristics of the clinical data of patients with systemic lupus erythematosus (SLE) complicated with liver failure, and to improve the cognition of the disease. The clinical data of patients with SLE complicated with liver failure hospitalized in Beijing Youan Hospital from January 2015 to December 2021 were collected retrospectively, including general information and laboratory examination data, and the clinical characteristics of the patients were summarized and analyzed. Twenty-one SLE patients with liver failure were analyzed. The diagnosis of liver involvement was earlier in 3 cases than that of SLE, and later in 2 cases. Eight patients were diagnosed with SLE and autoimmune hepatitis at the same time. The medical history is between 1 month and 30 years. This was the first case report of SLE complicated with liver failure. We found that: (1) among the 21 patients, organ cysts (liver and kidney cysts) were more common and the proportion of cholecystolithiasis and cholecystitis was higher than that in previous studies, but the proportion of renal function damage and joint involvement was lower. (2) The inflammatory reaction was more obvious in SLE patients with acute liver failure. The degree of liver function injury in SLE patients with autoimmune hepatitis was less than that in patients with other liver diseases. (3) The use of glucocorticoid in SLE patients with liver failure was worthy of further discussion.

**Table:** 

**Key Points** *• Patients with SLE complicated with liver failure have a lower proportion of renal impairment and joint involvement.* *• The study firstly reported SLE patients with liver failure.* *• Glucocorticoids in the treatment of SLE patients with liver failure are worthy of further discussion.*

**Key Points**

*• Patients with SLE complicated with liver failure have a lower proportion of renal impairment and joint involvement.*

*• The study firstly reported SLE patients with liver failure.*

*• Glucocorticoids in the treatment of SLE patients with liver failure are worthy of further discussion.*

## Introduction

Systemic lupus erythematosus (SLE) is one of the typical manifestations of autoimmune diseases. The global morbidity is 20–150/100,000 people [1], and the morbidity in China is about 100.3/100,000 people [2]. Most clinical features of SLE are multi-system damage with complex and diverse conditions, of which about 20% are accompanied with liver dysfunction [1]. As an important lymphoid organ, the liver participates in the immune response and is the target of autoimmune response [3]. Patients with SLE usually have mild liver dysfunction [4], while the advanced liver disease including cirrhosis and liver failure could be rarely found [5]. But there is no significant difference in the survival rate of SLE patients whether accompanied with liver dysfunction [4].

Clinically, most SLE with liver dysfunction is not only caused by SLE itself, but also caused by drug toxicity or other liver diseases, such as fat infiltration and overlapping autoimmune liver disease, virus infection, and alcoholism [6–11]. End-stage liver disease complicated with portal hypertension, cirrhosis, and hepatic encephalopathy is a rare complication of SLE unless there are nonalcoholic fatty liver disease (NAFLD), viral hepatitis, or autoimmune hepatitis (AIH) that existed simultaneously [3, 6]. It has been revealed that a few patients with end-stage liver disease, accompanied with portal hypertension and cirrhosis, did not die of liver failure, while the main cause of death was infection or cancer [4]. Runyon et al. have reported three death cases of patients with liver failure, suggesting that liver involvement in SLE patients is more common than recognized, and fatal liver diseases may occur [12].

This study firstly explored the clinical characteristics of liver failure in SLE patients, to provide some evidence for clinical diagnosis and treatment.

## Methods

### Patients/study design

This study is a retrospective study in a single center. We used the information retrieval system to screen all SLE patients hospitalized in Beijing Youan Hospital from January 2015 to December 2021. The hospitalization record of each patient could be queried in the medical record system by the case number (ID). The diagnosis of SLE was referred to “the guidelines for the diagnosis and treatment of systemic lupus erythematosus” issued by the rheumatology branch of the Chinese Medical Association in 2010 [13]. A total of 99 patients with SLE have been hospitalized in our hospital from 2015 to 2021. Among them, 21 patients under the guidelines for the diagnosis of liver failure with relatively complete clinical [14], serological, and radiological data were included. This study was approved by the Ethics Committee of Beijing Youan Hospital. As this study was retrospective, waiver of informed consent was requested at the ethics committee.

### Laboratory testing and imaging studies

Routine laboratory examinations include blood routine test, urine routine test, coagulation indexes, biochemical indexes of liver injury, biochemical indexes of renal injury, blood lipid series, autoantibody series, and immunoassay of special proteins. Abdominal CT examination recorded the appearance and size of the liver and spleen, parenchymal echo, bilateral signs of the gallbladder wall, gallbladder sound shadow, sound enhancement, stones, etc.

### Classification of liver failure

Liver failure is a serious liver damage caused by many factors, resulting in critical impairment or decompensation of synthesis, detoxification, metabolism, and biotransformation. It presents severe symptoms with jaundice, coagulation dysfunction, hepatorenal syndrome, hepatic encephalopathy, and ascites as the main clinical manifestations, resulting in high mortality. Generally, the major cause of liver failure in China is hepatitis virus.

Acute liver failure (ALF) is characterized by acute onset, grade II or above hepatic encephalopathy within 2 weeks, as well as the following manifestations: (1) extreme fatigue, accompanied with obvious anorexia, abdominal distension, nausea, vomiting, and other serious gastrointestinal symptoms; (2) jaundice deepens progressively in a short time, TBIL ≥ 10 upper limit of normal (ULN) or daily rise ≥ 17.1 μmol/L; (3) bleeding tendency, prothrombin activity (PTA) ≤ 40%, or INR ≥ 1.5, and other reasons are excluded; (4) progressive reduction of liver volume.

Subacute liver failure (SALF) is characterized by relatively acute onset as well as the following manifestations in 2–26 weeks: (1) extreme fatigue with obvious gastrointestinal symptoms; (2) jaundice deepens rapidly, TBIL ≥ 10 × ULN or daily rise ≥ 17.1 μmol/L; (3) with or without hepatic encephalopathy; (4) bleeding tendency, PTA ≤ 40%, or INR ≥ 1.5, and other reasons are excluded.

Acute-on-chronic liver failure (ACLF) is characterized by acute jaundice, coagulation dysfunction, and other liver failure symptoms triggered by various factors on the basis of chronic liver disease. It could be combined with hepatic encephalopathy, ascites, electrolyte disorder, infection, hepatorenal syndrome, hepatopulmonary syndrome, and other complications, as well as extrahepatic organ failure. Usually, the patient shows rapid deepening of jaundice with TBIL ≥ 10 × ULN or daily rise ≥ 17.1 μmol/L, hemorrhage, PTA ≤ 40% or INR ≥ 1.5.

Chronic liver failure (CLF) is characterized by slow progressive decline and decompensation of liver function on the basis of cirrhosis: (1) TBIL rises, usually < 10 × ULN; (2) albumin (ALB) decreased significantly; (3) platelet decreased significantly, PTA ≤ 40% or INR ≥ 1.5, and other reasons are excluded; (4) intractable ascites or portal hypertension; (5) hepatic encephalopathy.

### Data collection and follow-up

We obtain clinical data and quality indicators related to laboratory examination through the medical record system.

### Statistical analysis

SPSS 26.0 software was used to analyze the data. The mean values of data conforming to a normal distribution are expressed as mean ± SD or median (range); classified variables were indicated as frequency or ratio. The mean values of data that did not conform to normal distribution were expressed as median ± quartile. Comparison of results between groups for continuous data using Student’s *t*-test or Mann–Whitney *u* test (*p* < 0.05) was considered statistically significant.

## Results

### Characteristics of SLE patients with liver failure

In this study, 21 patients of SLE with liver failure were included, including 4 male patients (19.05%); the average age was 42.75 ± 10.54 years and 17 female patients (80.95%); and the average age was 48.12 ± 3.41 years. Among the 21 SLE patients with liver failure included in this study, there were 3 patients of drug-induced liver disease, 3 patients of non-basic liver disease, 5 patients of chronic hepatitis, 8 patients of AIH, and 1 patient of alcoholic liver disease; the medical history is between 1 month and 30 years. There were 3 patients diagnosed with liver involvement earlier than SLE, 2 patients later than that, 8 patients diagnosed with SLE and AIH at the same time, 5 patients combined with renal injury, and 3 patients with abnormal thyroid function. Clinical examination showed that ascites could be found in 12 patients, hyperammonemia/hepatic encephalopathy in 10 patients, splenomegaly in 9 patients, pleural effusion in 9 patients, and hydropericardium in 2 patients. Additionally, compared with previous studies, we found more patients with organ cyst, such as liver cyst in 5 patients, renal cyst in 7 patients, gallbladder stone in 5 patients, and cholecystitis in 8 patients. In urine examination, the protein of cases 7, 11, 13, and 20 was positive, and the occult blood of cases 3, 11, 13, 19, and 20 was positive. Officially, the liver biopsy is considered the gold standard for diagnosis of various liver diseases. The pathological results of liver biopsy revealed that case 9 had an acute attack of chronic hepatitis B (G2, S3-4), case 11 had massive necrosis of liver tissue which was consistent with the diagnostic criteria of ALF, case 18 had multi-hepatic lobular necrosis accompanied with hepatocyte regeneration, and case 20 had moderate hepatitis (G2) and liver cirrhosis. The results are shown in Table 1.

**Table 1:** Demographic features (*n* = 21)

	**Data**
Characteristic	
Mean age, years ± SD (range)	47.10 ± 15.14 (21–70)
Male, *n* (%)	4 (19.05%)
Female, *n* (%)	17 (80.95%)
Organ involvement, ***n*** (%)	
Renal	5 (23.81%)
Lung	0 (0%)
Thyroid dysfunction	3 (14.29%)
Neuropsychiatric	0 (0%)
Hematologic	1 (4.76%)
Serositis	9 (42.86%)
Hydropericardium	2 (9.52%)
Arthritis	2 (9.52%)
Complications, ***n*** (%)	
Splenomegaly	9 (42.86%)
Hepatic encephalopathy	10 (47.62%)
Portal hypertension	4 (19.05%)
Esophageal and gastric varices	3 (14.29%)
Ascites	12 (57.14%)
Hydrothorax	9 (42.86%)
Pleural thickening	8 (38.10%)
Gastrointestinal bleeding	1 (4.76%)
Cholecystitis	8 (38.10%)
Cholecystolithiasis	5 (23.81%)
Renal cyst	7 (33.33%)
Hepatic cyst	5 (23.81%)
Splenic cyst	1 (4.76%)
Combined liver diseases, ***n*** (%)	
Viral hepatitis	5 (23.81%)
HAV	1 (4.76%)
HBV	3 (14.28)
HCV	1 (4.76%)
Alcoholic liver disease	1 (4.76%)
AIH	8 (38.10%)
SLE itself liver injury	3 (14.28)
Classification of liver failure, ***n*** (%)	
ALF	5 (23.81%)
SALF	6 (28.57%)
ACLF	7 (33.33%)
CLF	3 (14.29%)

### Detection of antibody and immunoglobulin in serum

The auto-antibody examination was detected in 19 patients, of which ANA positive in 18 patients (94.7%), ACA positive in 4 patients (21.1%), anti-parietal cell antibody (APCA) positive in 2 patients (10.5%), anti-proliferating cell nuclear antigen antibody (PCNA) positive in 1 patient (5.3%), and anti-mitochondrial antibody (AMA) positive in 2 patients (10.5%). The ANA spectrum was detected in 18 patients, of which nRNP positive in 3 patients (16.7%), ACA-B positive in 3 patients (16.7%), ARPA positive in 3 patients (16.7%), anti-Ro-52 antibody positive in 3 patients (16.7%), SSA in 2 patients (11.1%), SSB in 1 patient (5.6%), anti-SM antibody positive in two patients (11.1%), dsDNA positive in 6 patients (33.3%), Jo-1 positive in 1 patient (5.6%), ANuA positive in 4 patients (22.2%), and AHA positive in 3 patients (16.7%). Moreover, the complement and immunoglobulin (Ig) were detected in twenty patients, of which C3 was 0.54 (0.172–2.46) decreased in 18 patients (90%), C4 was 0.27 (0.03–2.82) decreased in 13 patients (65%), IgG was 20.96 (1.22–53.70) increased in 15 patients (75%), and IgM was 1.71 (0.22–6.88) increased in 5 patients (25%). Since the detection value of IgA in 1 patient was lower than the lower limit, the mean value of other 19 patients was 3.74 (< 0.233–9.72), of which 5 patients increased (25%). The results are shown in Table 2.

**Table 2:** Antibody and immunoglobulin test

No	ANA	Ro-52	SSA	SSB	Sm	Scl-70	dsDNA	Jo-1	ANuA	Histone	nRNP	CENP B	ARPA	IgG	IgA	IgM	C3	C4
1	+	\	\	\	\	\	\	\	\	\	\	\	\	17.6	3.47	0.804	0.381	0.112
2	+	n	2 +	1 +	n	n	n	n	n	n	n	n	n	\	\	\	\	\
3	\	n	n	n	n	n	n	n	n	n	n	n	n	7.23	1.37	0.919	0.19	0.079
4	+	\	\	\	\	\	\	\	\	\	\	\	\	20.6	3.72	0.633	0.172	0.042
5	+	n	n	n	n	n	4 +	n	2 +	1 +	n	n	n	53.7	2.64	0.772	0.716	0.058
6	+	n	n	n	n	n	n	n	n	n	n	n	n	6.44	1.9	0.227	0.486	0.13
7	+	n	n	n	n	n	n	n	2 +	n	n	n	n	24	2.81	1.85	0.352	0.08
8	+	n	n	n	n	n	4 +	n	2 +	n	n	n	n	34.1	3.59	1.08	0.565	0.098
9	n	n	n	n	n	n	n	n	n	n	n	n	n	24	3.74	1.42	0.338	0.034
10		1 +	n	n	n	n	3 +	n	n	1 +	n	4 +	n	23.4	2.43	1.54	0.296	0.039
11	+	\	\	\	\	\	\	\	\	\	\	\	\	11.2	3.28	1.73	0.286	0.062
12	+	n	n	n	n	n	2 +	n	n	n	n	3 +	n	21.3	7.34	2.58	0.332	0.068
13	+	n	n	n	n	n	4 +	n	n	n	n	n	n	15.1	3.67	0.861	0.467	0.092
14	+	n	n	n	n	n	n	n	n	2 +	2 +	n	4 +	18.4	< 0.233	6.88	0.424	0.6
15	+	n	n	n	2 +	n	n	n	n	n	4 +	n	1 +	22.8	2.21	1.11	0.253	0.06
16	+	n	2 +	n	n	n	n	n	n	n	n	n	n	1.22	1.28	2.78	2.46	0.7
17	+	n	n	n	n	n	n	n	n	n	n	4 +	n	21.3	4.66	1.39	0.371	0.063
18	+	n	n	n	4 +	n	n	n	n	n	4 +	n	4 +	31.4	4.29	2.98	0.209	0.06
19	+	3 +	n	n	n	n	n	2 +	n	n	n	n	n	18.1	3.45	2.82	0.643	2.82
20	+	2 +	n	n	n	n	n	n	n	n	n	n	n	30.4	9.72	1.01	0.868	0.151
21	+	n	n	n	n	n	3 +	n	3 +	n	n	n	n	17	5.57	0.836	0.908	0.113

*ANA*, anti-nuclear antibody; *Ro-52*, anti-Ro-52 antibody; *SSA*, Sjögren’s syndrome antigen type A; *SSB*, Sjögren’s syndrome antigen type B; *Sm*, anti-Smith antibody; *Scl-70*, Scleroderma 70 antigen; *ds-DNA*, double-stranded DNA; *Jo-1*, anti-Jo-1 antibody; *ANuA*, anti-nucleosome antibody; *Histone*, anti-histone antibody; *nRNP*, anti-nuclear ribonucleoprotein; *CENP B*, anti-centromere antibody B; \, none detected; *n*, normal; + , positive. Reference value range of immunoglobulin: IgG (7–16), IgA (0.7–4), IgM, (0.4–2.3), C3 (0.9–1.8), C4 (0.1–0.4)

### Different types of liver failure in SLE patients

As shown in Table 3, the mean age of SLE patients with chronic liver failure was higher than that of the other three groups, but the difference was not statistically significant (*p* = 0.096). The hemoglobin (Hgb) of patients in the chronic liver failure group was 84.33 ± 12.34 g/L, which was mild anemia, lower than that of the other three groups and might be caused by cirrhosis. The platelet of patients in the ACLF group was fluctuated at 27 − 177 × 10^9^/L, lower than that of the other three groups. The liver function of patients in the acute liver failure group was the most seriously damaged, and the elevation of transaminase (ALT, AST, GGT), bilirubin (TBIL, DBIL), and ALP was most obvious compared with the other three groups, which might be related to the liver injury caused by acute inflammation. Meanwhile, the coagulation function of patients in the acute liver failure group was worse than that of the other three groups. And there was no significant difference in ALB among the four groups.

**Table 3:** Examination of SLE with different types of liver failure. Values are mean ± SD (range)

	ALF, *n* = 5	SALF, *n* = 6	ACLF, *n* = 7	CLF, *n* = 3	*p* value
Age, years	49.80 ± 11.52 (34–65)	36.83 ± 14.93 (21–56)	47.29 ± 15.23 (27–68)	62.67 ± 8.74 (53–70)	0.096
WBC (× 10^9^/L)	4.73 ± 3.03 (0.73–9.14)	4.46 ± 2.67 (2.37–9.59)	6.37 ± 3.82 (2.75–12.76)	7.37 ± 2.46 (4.82–9.74)	0.498
RBC (× 10^12^/L)	3.20 ± 0.86 (1.70–3.82)	3.47 ± 0.67 (2.55–4.36)	3.51 ± 0.95 (2.42–4.29)	2.94 ± 0.29 (2.60–3.12)	0.713
HGB (g/L)	106.00 ± 27.57 (60–132)	105.17 ± 20.13 (84–131)	111.57 ± 26.53 (80–140)	84.33 ± 12.34 (74–98)	0.445
PLT (× 10^9^/L)	116.60 ± 107.68 (54–308)	144.67 ± 74.32 (57–238)	101.14 ± 56.87 (27–177)	118.67 ± 99.02 (61–233)	0.817
ALT (U/L)	787.80 ± 1149.13 (138–2830)	309.83 ± 315.39 (31–856)	247.86 ± 130.10 (28–366)	25.00 ± 19.92 (13–48)	0.305
AST (U/L)	490.40 ± 392.95 (123–1155)	431.33 ± 326.30 (81–1008)	224.00 ± 183.75 (29–578)	38.67 ± 17.95 (25–59)	0.127
TBIL (μmol/L)	316.60 ± 116.44 (135.9–459.3)	231.07 ± 110.25 (69.80–362)	204.09 ± 186.16 (35.10–573.20)	35.87 ± 39.44 (12.60–81.40)	0.085
DBIL (μmol/L)	199.26 ± 83.54 (104.20–333)	175.27 ± 86.09 (50.10–302.30)	142.24 ± 133.50 (19.10–406.20)	21.67 ± 24.92 (6.00–50.40)	0.132
GGT (U/L)	485.60 ± 920.08 (39–2131)	183.83 ± 125.09 (65–375	96.43 ± 41.49 (19–160)	19.33 ± 7.51 (12–27)	0.440
ALP (U/L)	587.40 ± 1042.87 (68.00–2452.00)	217.17 ± 108.36 (110.00–407.00)	112.29 ± 51.59 (55.00–202.00)	83.33 ± 8.39 (78.00–93.00)	0.411
TBA(μmol/L)	162.46 ± 11.58 (147.20–176.70)	173.90 ± 51.96 (95.50–229.40)	140.83 ± 86.13 (6.70–238.70)	87.03 ± 66.27 (44.70–163.40)	0.278
ALB (g/L)	27.32 ± 3.29 (23.30–31.80)	29.63 ± 6.02 (24.40–40.70)	28.60 ± 2.13 (24.70–31.00)	27.17 ± 2.64 (25.40–30.20)	0.739
Cr (μmol/L)	72.20 ± 40.58 (45–140)	56.33 ± 16.33 (31–78)	49.86 ± 6.70 (41–59)	71.00 ± 13.89 (62–87)	0.319
BUN (mmol/L)	6.83 ± 6.23 (2.28–16.70)	3.99 ± 1.33 (2.87–6.50)	4.36 ± 1.09 (3.44–5.94)	11.17 ± 4.16 (6.62–14.79)	0.039
UA (mmol/L)	177.20 ± 77.15 (98–304)	253.83 ± 119.25 (139–484)	206.29 ± 109.99 (38–335)	437.00 ± 109.49 (334–552)	0.020
PT (S)	38.28 ± 26.31 (17.80–82.40)	27.97 ± 11.35 (17.40–43.30)	23.31 ± 8.18 (16.40–40.40)	22.73 ± 1.16 (21.40–23.50)	0.363
PTA (%)	29.40 ± 17.57 (9.00–53.00)	35.00 ± 16.24 (17.00–52.00)	40.86 ± 13.16 (19.00–59.00)	37.00 ± 2.00 (35.00–39.00)	0.614
INR (INR)	3.39 ± 2.35 (1.59–7.36)	2.46 ± 0.98 (1.55–3.71)	2.05 ± 0.68 (1.46–3.47)	2.01 ± 0.08 (1.92–2.05)	0.352
FIB (g/L)	1.73 ± 1.13 (0.79–3.64)	1.40 ± 0.26 (0.83–1.67)	1.98 ± 1.98 (0.72–6.42)	2.68 ± 1.05 (2.02–3.89)	0.609
TG (mmol/L)	1.77 ± 0.78 (1.09–2.98)	2.64 ± 1.72 (0.93–5.14)	1.05 ± 0.33 (0.73–1.51)	0.73 ± 0.16 (0.60–0.90)	0.038
CHOL (mmol/L)	4.44 ± 3.80 (2.28–11.20)	3.35 ± 0.73 (2.32–4.50)	2.46 ± 0.41 (1.79–2.91)	2.60 ± 0.42 (2.27–3.08)	0.356
HDL-C (mmol/L)	0.20 ± 0.17 (0.05–0.48)	0.27 ± 0.27 (0.04–0.73)	0.35 ± 0.32 (0.01–0.921)	0.69 ± 0.29 (0.39–0.97)	0.116
LDL-C (mmol/L)	3.17 ± 4.72 (0.82–11.60)	1.43 ± 0.48 (1.01–2.09)	1.41 ± 0.50 (0.42–1.98)	1.35 ± 0.25 (1.09–1.58)	0.576
C3 (g/L)	0.80 ± 0.94 (0.29–2.46)	0.36 ± 0.12 (0.21–0.49)	0.40 ± 0.20 (0.17 ± 0.72)	0.71 ± 0.31 (0.35–0.91)	0.424
C4 (g/L)	0.74 ± 1.20 (0.039–2.82)	0.09 ± 0.31 (0.06–0.13)	0.14 ± 0.20 (0.03–0.60)	0.11 ± 0.36 (0.08–0.15)	0.312

*WBC*, white blood cell count; *RBC*, red blood cell count; *Hgb*, serum hemoglobin; *PLT*, platelet count; *ALT*, alanine aminotransferase; *AST*, aspartate aminotransferase; *TBIL*, total bilirubin; *DBIL*, direct bilirubin; *GGT*, serum γ-glutamyltransferase; *ALP*, serum alkaline phosphatase; *TBA*, total bile acids; *ALB*, serum albumin; *Cr*, creatinine; *BUN*, blood urea nitrogen; *UA*, uric acid; *PT*, prothrombin time; *PTA*, prothrombin activity; *INR*, international normalized ratio; *FIB*, fibrinogen; *TG*, triglyceride; *CHOL*, total cholesterol; *HDL-C*, high-density lipoprotein cholesterol; *LDL-C*, low-density lipoprotein cholesterol; *C3*, complement 3; *C4*, complement 4

### The influence of AIH on laboratory examination to SLE patients

As shown in Table 4, there is no significant difference in age distribution between patients with or without AIH. The Hgb of patients with AIH was lower than that of patients without AIH. The PLT in patients with AIH decreased significantly, but there was no significant difference due to the small sample capacity. The liver function of patients without AIH was more serious, and the obvious elevation of transaminase (ALT, AST, GGT), bilirubin (TBIL, DBIL), and ALP might be related to the more grievous liver failure caused by other reasons than by AIH. In addition, the coagulation function of patients without AIH was worse than that of patients with AIH.

**Table 4:** Examination of SLE with or without AIH. Values are mean ± SD (range)

	With AIH, *n* = 8	Others, *n* = 13	*p* value
Age	47.46 ± 13.56 (27–70)	46.50 ± 18.42 (21–65)	0.096
WBC (× 10^9^/L)	4.79 ± 2.55 (2.37–9.74)	6.06 ± 3.46 (0.73–12.76)	0.381
RBC (× 10^12^/L)	3.19 ± 0.66 (2.42–4.36)	3.44 ± 0.83 (1.70–4.69)	0.480
HgB (g/L)	98.75.17 ± 20.53 (74–131)	108.08 ± 25.49 (60–140)	0.394
PLT (× 10^9^/L)	88.00 ± 49.31 (40–202)	139.31 ± 86.20 (27–308)	0.143
ALT (U/L)	178.33 ± 63.05 (13–452)	475.70 ± 740.21 (14–2830)	0.282
AST (U/L)	245.63 ± 213.70 (29–513)	366.08 ± 351.57 (25–1155)	0.395
TBIL (μmol/L)	156.61 ± 121.32 (12.60–330.40)	250.21 ± 165.81 (13.60–573.20)	0.184
DBIL (μmol/L)	102.88 ± 77.54 (8.60–210.60)	175.82 ± 119.17 (6.00–406.20)	0.141
GGT (U/L)	124.63 ± 133.20 (12–375)	251.31 ± 567.08 (19–2131)	0.545
ALP (U/L)	167.00 ± 117.39 (55.00–407.00)	303.08 ± 647.35 (68.00–2452.00)	0.567
TBA (μmol/L)	147.31 ± 65.70 (44.30–206.80)	148.01 ± 66.89 (6.70–238.70)	0.982
ALB (g/L)	29.23 ± 4.96 (24.40–40.70)	27.87 ± 2.94 (23.30–31.80)	0.439
Cr (μmol/L)	57.25 ± 16.93 (31–87)	61.77 ± 26.42 (41–140)	0.672
BUN (mmol/L)	6.56 ± 4.52 (2.69–14.79)	5.36 ± 3.89 (2.28–16.70)	0.528
UA (mmol/L)	319.75 ± 148.07 (151–552)	200.46 ± 96.63 (38–335)	0.037
PT (S)	24.59 ± 8.58 (17.40–41.20)	30.30 ± 18.04 (16.40–82.40)	0.415
PTA (%)	37.63 ± 11.57 (19.00–52.00)	34.85 ± 15.56 (9.00–59.00)	0.673
INR (INR)	2.17 ± 0.74 (1.55–3.58)	2.67 ± 1.60 (1.46–7.36)	0.414
FIB (g/L)	1.48 ± 0.46 (0.79–2.12)	2.09 ± 1.62 (0.72–6.42)	0.318
TG (mmol/L)	1.83 ± 1.64 (0.68–5.14)	1.41 ± 0.69 (0.60–2.98)	0.438
CHOL (mmol/L)	3.01 ± 0.70 (2.33–4.50)	3.32 ± 2.43 (1.79–11.20)	0.731
HDL-C (mmol/L)	0.36 ± 0.28 (0.07–0.97)	0.34 ± 0.32 (0.01–0.92)	0.887
LDL-C (mmol/L)	1.39 ± 0.36 (1.01–2.09)	2.16 ± 3.01 (0.42–11.60)	0.484
C3 (g/L)	0.40 ± 0.22 (0.21–0.91)	0.63 ± 0.61 (0.17–2.46)	0.329
C4 (g/L)	0.72 ± 0.22 (0.04–0.11)	0.41 ± 0.79 (0.03–2.82)	0.251

## Discussion

Patients with SLE associated with liver dysfunction are more common clinically. Currently published articles mainly include research studies on SLE with liver dysfunction [4, 15], SLE with PBC [16], cirrhosis [17], and case reports [18]. However, no serious patients of combined liver failure have been found [19]. We firstly analyzed 21 patients of SLE patients with liver failure and compared the clinical manifestations of SLE complicated with different types of liver failure. However, this is a small-scale retrospective study; the clinical data was limited and insufficient.

Among the 21 SLE patients with liver failure included in this study, there were 3 patients of drug-induced liver disease (14.29%), 3 patients of non-basic liver disease (14.29%), 3 patients of chronic hepatitis B (14.29%), 1 patient of chronic hepatitis C (4.76%), 8 patients of AIH (38.10%), and 1 patient of alcoholic liver disease (4.76%); the medical history is between 1 month and 30 years. In previous cohort studies, NAFLD and chronic hepatitis C were the most common reasons of liver dysfunction [6], which was different from the results of this study. Among the 21 patients included in this study, 2 died of ineffective treatment, and the other patients were discharged from hospital after recovery. Compared with previous studies [5, 6], the age of 21 patients was roughly similar (Table 1). The incidence rate of splenomegaly in this study was 42.86% (9 patients), which was significantly higher than that of Chowdhary et al. (12%) [6]. Organ cysts were common in our study, including 5 patients of liver cysts (23.81%), 7 patients of renal cysts (33.33%), 5 patients of gallbladder stones (23.81%), and 8 patients of cholecystitis (38.10%). However, no patients of organ cysts were found in previous studies. The patients with impaired renal function accounted for 23.81% and with arthritis accounted for 9.53%, which were lower than the proportion of renal involvement and arthritis in previous studies [6, 12]. In this study, the urine protein of 4 patients (cases 7, 11, 13, 20) was positive. The measurement of albuminuria has significance in evaluating the disease activity of patients with renal involvement. Early renal manifestations could be judged by the reduction of albuminuria [20]. The patients with serositis accounted for 42.86%, which was similar to 37% of the results of Chowdhary et al.’s studies [6], and lower than 79% that reported by Runyon et al. [12]. Among 193 SLE patients reported by Suzuki et al., 3 patients (1.6%) had gallstones and/or cholecystitis, while in our study, 5 patients (23.81%) had gallstones and 8 patients (38.10%) had cholecystitis, which was significantly higher than that in previous studies. It was considered that the reason might be related to SLE patients with liver failure were mostly in end-stage liver disease with more complications. Moreover, the impairment of liver function and coagulation function was more severe in patients with non-autoimmune liver disease than patients with autoimmune liver disease (Table 4), but there was no statistical significance due to the small sample capacity. In this study, ANA was detected in 19 patients, of which 18 were positive, anti-dsDNA was detected in 18 patients, of which 6 were positive, and anti-Sm antibody was positive in 2 patients, as shown in Table 2. Several studies investigated that more than 25% of people were ANA positive even if without rheumatic disease or other ANA-related diseases. Meanwhile, anti-dsDNA and anti-Sm antibodies are highly specific for SLE. The patient of case 21 had positive dsDNA antibody and renal insufficiency. Previous studies suggested that anti-dsDNA antibody might be associated with SLE activity, especially in patients with nephritis [6]. 9.7% (17/175) of patients became negative for ANA within 53.5 months, which was related to the reduction of the risk of SLE reoccurrence in the future [21]. The patients of case 4 and case 9 had HBV-related ACLF, in which the ANA of case 4 was positive, but case 9 was negative. It has been previously reported that ANA positive is common in HBV-associated ACLFs and does not seem to be associated with adverse outcomes [22]. The sample size of this study is small, which needs to be confirmed by further research with larger sample size.

It has been found in this study the level of GGT in patients with ALF (485.60 ± 920.08 U/L) > SALF (183.83 ± 125.09 U/L) > ACLF (96.43 ± 41.49 U/L) > CLF (19.33 ± 7.51 U/L). And the GGT level of patients with AIH (124.63 ± 133.20 U/L) was lower than that of patients with other liver diseases (251.31 ± 567.08 U/L). As is well known, GGT has a strong pro-inflammatory effect [23]. Considering the inflammatory response is more obvious in patients with acute liver failure and other liver diseases, high level of GGT might be a significant risk factor for the aggravation of SLE [23]. Therefore, with the increase of GGT level, the risk of SLE aggravation ascends accordingly. Additionally, we also found the level of TBIL in patients with ALF (316.60 ± 116.44 μmol/L) > SALF (231.07 ± 110.25 μmol/L) > ACLF (204.09 ± 186.16 μmol/L) > CLF (35.87 ± 39.44 μmol/L). And the TBIL level of patients with AIH (156.61 ± 121.32 μmol/L) was lower than that of patients with other liver diseases (250.21 ± 165.81 μmol/L). TBIL has the same anti-inflammatory effect with GGT [23], which proves that the inflammatory response in patients with acute liver failure and other liver diseases is more serious from different aspects.

The complement was detected in 20 patients in this study. The mean value of C3 was 0.54 (0.172–2.46), of which 18 patients (90%) were decreased, and the mean value of C4 was 0.27 (0.03–2.82), of which 13 patients (65%) were decreased. It was consistent with the previous study that the incidence rates of simultaneous reduction of C3 and C4 levels in SLE patients (73.42%) were significantly higher than those in non-SLE patients (*p* < 0.01) [24]. The incidence of low total C3 level (79.75%), low total C4 level (84.18%), and low total complement level (C3 or C4) (90.51%) in SLE patients was higher than that in non-SLE patients (*p* < 0.01) [24]. The decrease of C3 level was one of the most important risk factors for cytopenia and mucosal skin lesions, so the activity of SLE might be negatively correlated with C3 level [25]. Meanwhile, low levels of C3 and C4 could predict the recurrence of SLE [25]. Previous studies showed the C3 level in HBV-ACLF (6568 μg/mL) < CHB group (8916 μg/mL) < control group (15,653 μg/mL); the difference was statistically significant (*p* < 0.05). Previous articles reported that low level of C3 suggested worse prognosis [26], and the levels of C3 and C4 in the severe group were significantly lower than those in the non-severe group [23]. In this study, the C3 level of patients with subacute liver failure was lower than that of the other three groups, and the C3 level of AIH patients was lower than that of patients with other liver diseases. It is relevant to consider that the patient is in the active stage of SLE disease.

Glucocorticoid is a powerful anti-inflammatory and immunosuppressive drug, which could effectively inhibit the immune system [27], and has been widely used in the treatment of patients with SLE [28] and liver failure [29]. Up to 80% of SLE patients have used glucocorticoid, and most of them have received long-term treatment [30, 31]. Furthermore, high-dose shock therapy could also be used in patients with lupus crisis [32]. However, the high-dose glucocorticoid treatment was not recommended for patients with liver failure, especially patients with liver cirrhosis [29]. Another report demonstrated patients with mild liver failure, high risk of rapid disease progression, and low HE grade and MELD score, but high ALT level had the best response to glucocorticoid treatment [33]. Therefore, the diagnosis and treatment of SLE patients with liver failure in critical condition has become a new challenge.

## Conclusion

This was the first series case reports on SLE patients with liver failure. We found that SLE patients complicated with organ cysts (liver and kidney cysts) were more common and the proportion of cholecystolithiasis and cholecystitis was higher than that in previous studies, but the proportion of renal function damage and joint involvement was lower. The inflammatory reaction was more obvious in SLE patients with acute liver failure. The degree of liver function injury in SLE patients with AIH was less than that in patients with other liver diseases. The use of glucocorticoid in SLE patients with liver failure was worthy of further discussion.

To sum up, there were several limitations in our study. Due to the small sample capacity and only a few laboratory examination items, there were differences between the four groups, but no statistical significance. Moreover, the patients were not followed up for a long time in this retrospective study. In general, more research is urgently needed to explore the characteristics and mechanism of SLE patients with liver failure in order to improve the prognosis.

## Data Availability

The datasets used and analyzed during the current study are available from the corresponding author on reasonable request.
